# Enhancement on selenium volatilization for phytoremediation: role of plant and soil microbe interaction

**DOI:** 10.3389/fpls.2024.1504528

**Published:** 2024-12-23

**Authors:** Ranju R. Karna, Samantha T. Kumara, Vance J. McCracken, Thomas J. Fowler, Zhi-Qing Lin

**Affiliations:** ^1^ Department of Environmental Sciences, Southern Illinois University Edwardsville, Edwardsville, IL, United States; ^2^ Department of Biological Sciences, Southern Illinois University Edwardsville, Edwardsville, IL, United States

**Keywords:** phytoremediation, selenium, volatilization, plant and microbial interaction, rabbitfoot grass, Indian mustard, *Bacillus cereus*

## Abstract

This study aimed at quantifying the potential effects of plant and soil microbial interaction on selenium (Se) volatilization, with the specific objectives of identifying soil bacteria associated with rabbitfoot grass (*Polypogon monspeliensis*) and demonstrating the enhancement of Se volatilization in the soil-Indian mustard (*Brassica juncea*) system through inoculation of the soil with the identified best Se-volatilizing bacterial strain. Soil bacteria were isolated from topsoil and rhizosphere soils of rabbitfoot grass, and the bacterial colonies were characterized via PCR-DGGE and DGGE band analysis prior to their identification using 16S rDNA sequencing technique. *Bacillus cereus* produced over 500-fold more volatile Se in a culture medium treated with 15 µg Se/mL (equal mixture of SeO_4_^2-^, SeO_3_^2-^ and selenomethionine) than any of the other eight identified bacterial strains. Inoculation of Indian mustard vegetated soil with the best Se volatilizing bacterial strain *B. cereus* resulted in a significant (p<0.05) increase in Se volatilization during a 7-day time period, compared to the soil-plant system without inoculation of *B. cereus*. Thus, inoculation of the soil with *B. cereus* substantially enhanced Se removal via biogenic volatilization in the soil-Indian mustard system. This study evaluated the role of *B. cereus* in enhancing Se volatilization in soil-plant systems, and demonstrated the importance of plant and soil microbial interaction for Se phytoremediation.

## 1 Introduction

Selenium (Se) is an important naturally occurring metalloid element required in trace amounts by humans and animals for the formation of amino acids such as selenocysteine (SeCys) and selenomethionine (SeMet). Selenocysteine is a component of some important selenoproteins such as glutathione peroxidase, selenoprotein-P, thioredoxin reductase, and deiodinases, and therefore, plays an important role in mitigating oxidative damages, redox protection, regulation of inflammation, and thyroid hormone metabolism (Schwarz and Foltz, 1957; Klein, 2004; Gärtner, 2009). However, excessive Se concentrations in water, soils, and plants may result in Se poisoning of animals and humans, causing developmental disorders and damage to skin, gastrointestinal and nervous systems (Bañuelos et al., 2005a; Navarro-Alarcon and Cabrera-Vique, 2008; Ackerman and Eagles-Smith, 2009). One notable example is the environmental disaster at the Kesterson reservoir in the San Joaquin Valley, California, where bioaccumulation of Se from the contaminated agricultural drainage water resulted in the deformity and death of fish and birds (Ohlendorf et al., 1986).

High levels of Se occur naturally in certain Cretaceous shale sediments and soils, and Se contamination from industrial sources is also widespread, occurring in one-third of the hazardous waste Superfund sites listed on the U.S. National Priority List. Various remediation technologies based on biological, chemical, and physical methods have been proposed for removal of Se from contaminated environments. Phytoremediation technologies use plants and soil microbes to remove, stabilize, or detoxify pollutants in soil and water, and thus, have become an environmentally sustainable and cost-competitive remediation approach (Chaney et al., 1997; Dungan and Frankenberger, 1999; Lin and Terry, 2003). Among the phytotechnology-based approaches, biogenic volatilization is considered one of the more promising methods for Se remediation (Bañuelos et al., 1996; Lin, 2019; Wang et al., 2024). Biogenic volatilization removed 5-10% of the total annual Se input in a constructed treatment wetland (Lin et al., 2002; Lin and Terry, 2003). Similarly, about 30% of the Se mass removed by a treatment wetland located adjacent to the San Francisco Bay was volatilized into the atmosphere (Hansen et al., 1998). Volatile Se compounds will be oxidized and transformed into aerosols in the atmosphere, and airborne Se can further deposit to ground surfaces via atmospheric wet and dry deposition (Lin, 2019). The input of airborne Se is generally beneficial to humans and animals as an essential nutrient element. The biogenic volatilization process in phytoremediation minimizes biomagnification of toxic Se accumulated in food chains; efficient biogenic volatilization is therefore valuable for the development of an environmentally sound and cost-competitive phytoremediation technology for remediating Se-contaminated environments under field conditions (Lin and Terry, 2003).

Effective biogenic Se volatilization generally requires a plant species with superior ability to metabolize inorganic Se into volatile organic Se compounds such as dimethylselenide (DMSe) and dimethyldiselenide (DMDSe) (Terry et al., 2000; Lin, 2019). Earlier studies showed that rabbitfoot grass (*Polypogon monspeliensis*) in treatment wetlands is a particularly efficient Se volatilizer (Lin et al., 2000; Lin and Terry, 2003); rabbitfoot grass could transfer 35-49% of total monthly Se mass input to the atmosphere in a constructed wetland (Lin and Terry, 2003). In the San Joaquin Valley of central California, the presence of *Salicornia bigelovii*, a halophytic plant species, could also remove 155 ± 25 µg Se/m^2^/day from a contaminated field (Lin et al., 2000). Similarly, a study conducted in the Kesterson Reservoir in central California reported removal of 68-88% Se from a contaminated site over an eight-year time period via microbial Se volatilization (Flury et al., 1997). Irrespective of the independent contribution of plants and soil microbes in Se volatilization, the interaction between plant and soil microbes is considered to play significant roles in this biological methylation process (Terry et al., 2000). One of the major factors affecting Se volatilization might be due to contributions by plant-associated soil microbes. However, the plant-associated soil microbes responsible for high Se volatilization in the soil-rabbitfoot grass system need to be further characterized.

Indian mustard (*Brassica juncea*) has long been identified as a good plant species for Se phytoremediation (Li and Liu, 2022). The plant has also been genetically engineered to further enhance Se accumulation and volatilization. A field trial showed that transgenic Indian mustard accumulates more than double the amount of Se compared to wild type, but no significant increase in Se volatilization (Bañuelos et al., 2005b). In this study, we hypothesized that when a soil-Indian mustard system is inoculated with the identified rabbitfoot grass-associated soil bacterial strain with superior ability to volatilize Se, the rate of Se volatilization in the soil-Indian mustard system would be significantly enhanced due to the addition and establishment of inoculated bacterial population in the soil-plant system. Thus, the specific objectives of this study were to (1) evaluate impacts of soil Se on the bacterial community in the soil-rabbitfoot grass system, (2) identify the soil bacterial strains with the best ability to volatilize Se in the soil-rabbit foot grass system, and (3) determine rates of Se volatilization in the soil-Indian mustard system after inoculation of the best Se volatilizing bacterial strain to the soil.

## 2 Materials and methods

### 2.1 Effects of Se on soil bacterial community

#### 2.1.1 Cultivation of rabbitfoot grass

Six pots (9-cm in height and 10-cm in diameter) were prepared in a greenhouse, with each pot containing 500 g (dry weight, DW) local agricultural clay loam soil mixed with commercial fine sand (3:1, v/v). Rabbitfoot grass (*Polypogon monspeliensis* (L.) Desf.) seeds were purchased from Cook’s Garden Inc. (Londonderry, Vermont) and germinated in four of the six pots. Two of the vegetated pots were treated with 5 mg/kg Se (Na_2_SeO_4_), and two bare soil pots were used as control. All pots were irrigated with 50 ml tap water every other day, and the photoperiod was 14 hours.

Upon pre-maturation (prior to the flowering stage) of rabbitfoot grass at approximately four weeks, three soil samples were randomly collected from both topsoil and rhizosphere of rabbitfoot grass in each vegetated pot. Bulk soil samples were also collected from the surface and the middle (similar to the root zone depth) of the bare soil pots. The rhizosphere soil was collected directly from plant root surfaces within about 1-2 mm thin layer. The soil samples with roots were transferred in 10 ml phosphate-buffered saline solution (PBS, Mediatech, Manassas, VA), vortexed in order to detach soil from root hairs, and centrifuged at 10,000 x g at 4 °C for 10 min. The topsoil samples were treated following a similar approach. Soil pellets were stored at -80°C for subsequent microbial analysis.

#### 2.1.2 Microbial culture

One g of topsoil or rhizosphere soil sample was added to 9 ml nutrient broth (NB; Difco, Becton Dickinson, Sparks, MD), vortexed, and ten-fold serial dilutions prepared ranging from 10^-1^ to 10^-6^. For each dilution 100 µl was spread onto each of five separate nutrient agar (Difco) plates and incubated overnight at 37 °C. Representative colonies of different sizes, shapes, and colors were streaked for further isolation on fresh nutrient agar plates, and again incubated overnight to ensure the purity of cultures. Glycerol stocks of pure cultures were prepared from overnight nutrient broth cultures and stored at -80 °C.

#### 2.1.3 DNA extraction, amplification, and denaturing gradient gel electrophoresis

Bacterial DNA was extracted from the soil samples using a PowerSoil^®^ DNA Kit (Mo-Bio Laboratories, CA), which has been shown to effectively lyse various microbes in soil while decreasing concentrations of PCR-inhibiting substances found in soil, such as humic acids (Whitehouse and Hottel, 2007; Alcántara-Hernández et al., 2009; Giubilei et al., 2009). Mechanical lysis (bead-beating) followed by phenol-chloroform extraction was performed to extract DNA from pure cultures (Stahl et al., 1988). Extracted DNAs were quantified using a Nanodrop spectrophotometer (Thermo Scientific, Wilmington, DE) and their purity was assessed by agarose gel electrophoresis.

DNA was amplified using primers specific for conserved sequences flanking the variable V3 region of the bacterial 16S rDNA molecule (Muyzer et al., 1996). Each reaction contained 150 ng of target DNA, 5 µl of 10 X Ex-Taq buffer (TaKaRa Shuzo, Otsu, Japan), 0.2 mM dNTPs, 500 nM each of forward primer (GC-314F; 5’CGCCCGCCGCGCGCGGCGGGCGGGGGGGGCACGGGGGGCCTACGGGAGGCAG-CAG 3’) and reverse primer (534R; 5’ATTACCGCGGCTGCTGG3’), and 5 U of Hot Start TaKaRa Taq Polymerase. The forward primer contains 40 base pair (bp) of high G+C content (a GC clamp), which prevents complete dissociation of the DNA strands during DGGE (Muyzer et al., 1996). The final volume of PCR mixtures was adjusted to 50 µl by adding ultra-pure water.

To reduce spurious PCR products, touchdown PCR was performed (Muyzer et al., 1996). PCR conditions included an initial denaturing step at 95 °C for 15 min (for enzyme activation and target denaturation), followed by melting at 95 °C for 1 min, annealing at 65 °C for 45 sec (decreased by 0.5 °C each cycle), and extension at 72 °C for 1 min, for a total of 20 cycles. Ten additional cycles of 95 °C for 1 min, 55 °C for 45 sec, and 72 °C for 1 min were performed, followed by a final extension of 5 min at 72 °C. The final approximately 200 base pair product was confirmed by electrophoresis on a 3% agarose gel with ethidium bromide staining.

#### 2.1.4 Denaturing gradient gel electrophoresis (DGGE) and DGGE band analysis

DGGE was performed using 8% (wt/vol.) polyacrylamide gels (acrylamide/bisacrylamide ratio, 37.5:1) containing a linear chemical gradient of 35-70% DNA denaturants (100% denaturant is equivalent to 7M urea and 40% deionized formamide). Gradients were formed using a GM-40 gradient former (CBS Scientific, Del Mar, CA) connected to a peristaltic pump. Following loading of 1:1 mix of sample or 1 kb ladder standard with 2X loading dye, gels were electrophoresed at 58 °C for 8 h at 100 V in the D-Code Universal Mutation Detection System (Bio-Rad Laboratories Ltd., Hercules, California). Following staining in GelStar^®^ (Lonza, Rockland, ME), and photographing of gels, unique or differentially expressed bands from the different treatment and control groups were excised from the DGGE gel under minimum ultraviolet ray exposure. Excised bands were washed in 10 µl of sterile MQ water and vortexed at high speed with 0.25 g of sterile glass beads (0.1 mm diameter) to elute DNA from bands. Eluted DNA was stored at -80 °C for cloning and sequencing.

DGGE band analysis was conducted using Quantity One software (Bio-rad, version 4.6.6) in order to determine the relative front (RF) value for each band following the protocol provided. Briefly, RF value is calculated by measuring the distance of the band from the top of its defined lane and dividing by the total length of the lane. In this experiment we determined the normalized relative front (NRF) that is determined based on a comparison of the relative front for the bands of interest to the relative front of the bands in the standard. At least two lanes containing standards (1 kb DNA ladder; Invitrogen, Carlsbad, CA) were used for each DGGE gel. Determination of NRF values allowed gel-to-gel comparisons. DGGE gel bands with differing NRF values and pure isolates were selected for cloning and sequencing.

**Figure 1:**
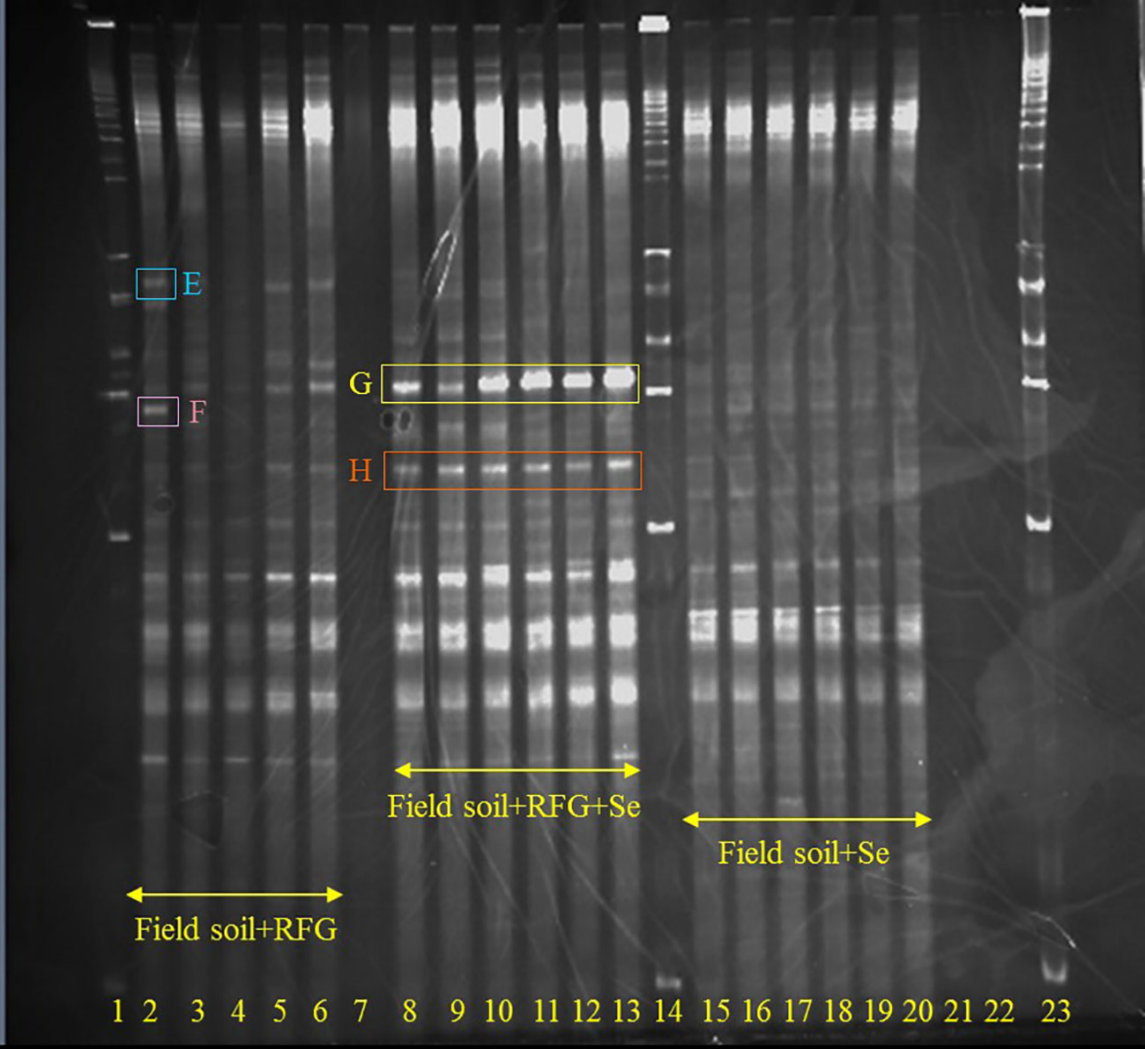
Effects of Se on the microbial community in the rhizosphere of a soil-rabbitfoot grass system. DGGE analysis of 16S rDNA fragments amplified from rhizosphere DNA taken from the rabbitfoot grass soil without Se treatment (lanes 2 to 6), the rabbitfoot grass soil with Se treatment (lanes 8 to 13), and Se-contaminated soil (lanes 15-20). Lanes 1, 14 and 23: 1 kb ladders. Bands excised, cloned, sequenced and identified as: E, *Niastella* sp., F, Uncultured Bacterium Clone; G, Xanthomonadales; H, Uncultured Alphaproteobacterium Clone.

To assess the impact of Se on microbial populations in the soil-rabbitfoot grass system, DGGE profiles for the soil-rabbitfoot grass treated with Se, and the soil-rabbitfoot grass not treated with Se were also compared using Sorensen’s index to determine the similarity and differences in the banding pattern between microbial communities associated with the rabbitfoot grass soil with Se treatment and the rabbitfoot grass soil without Se treatment. The DGGE band image was taken using Bio-Rad Gel-Doc XR documentation system, and no enhancement was made on the image (**Figure 1**). Sorensen’s index is a pairwise similarity coefficient CS, which was determined by CS= (2j/a+b)*100, where a is the number of DGGE bands in lane 1, b is the number of DGGE bands in lane 2, and j is the number of common bands (Magurran, 1988; La Valley et al., 2009).

#### 2.1.5 Cloning and sequencing of pure isolates and DGGE bands

The 16S rDNA gene from several pure isolates and from organisms differentially detected in DGGE gels were cloned and sequenced. For sequencing of isolated strains, a nearly full-length fragment of the 16S rDNA was amplified with primers: 8FE (forward primer): 5’ AGAGTTTGATCMTGGCTCAG 3’ and 1492R (reverse primer): GGMTACCTTGTTACGACTT 3’ (Salzman et al., 2002). PCR mix was prepared as described above, but only 50 ng of DNA were added for each reaction. PCR of full length 16S rDNA for pure culture isolates used an initial denaturing step at 95 °C for 15 min, followed by 95 °C for 1 min, 60 °C for 45 sec, and 72 °C for 1 min for a total of 29 cycles with a final extension of 5 min at 72 °C. The final product length of 1500 bp was confirmed on a 1% agarose gel with ethidium bromide staining.

Two µl of DNA eluted from the excised DGGE bands were used as target DNA for a subsequent PCR amplification with primers 341F (without GC clamp) and 534R. The initial denaturing step was at 95 °C for 15 min (for enzyme activation and target denaturation), followed by 95 °C for 1 min, 60 °C for 45 sec, and 72 °C for 1 min, for a total of 29 cycles with a final extension of 5 min at 75 °C. The final product length of approximately 200 bp was confirmed on a 3% agarose gel followed by ethidium bromide staining.

**Figure 2:**
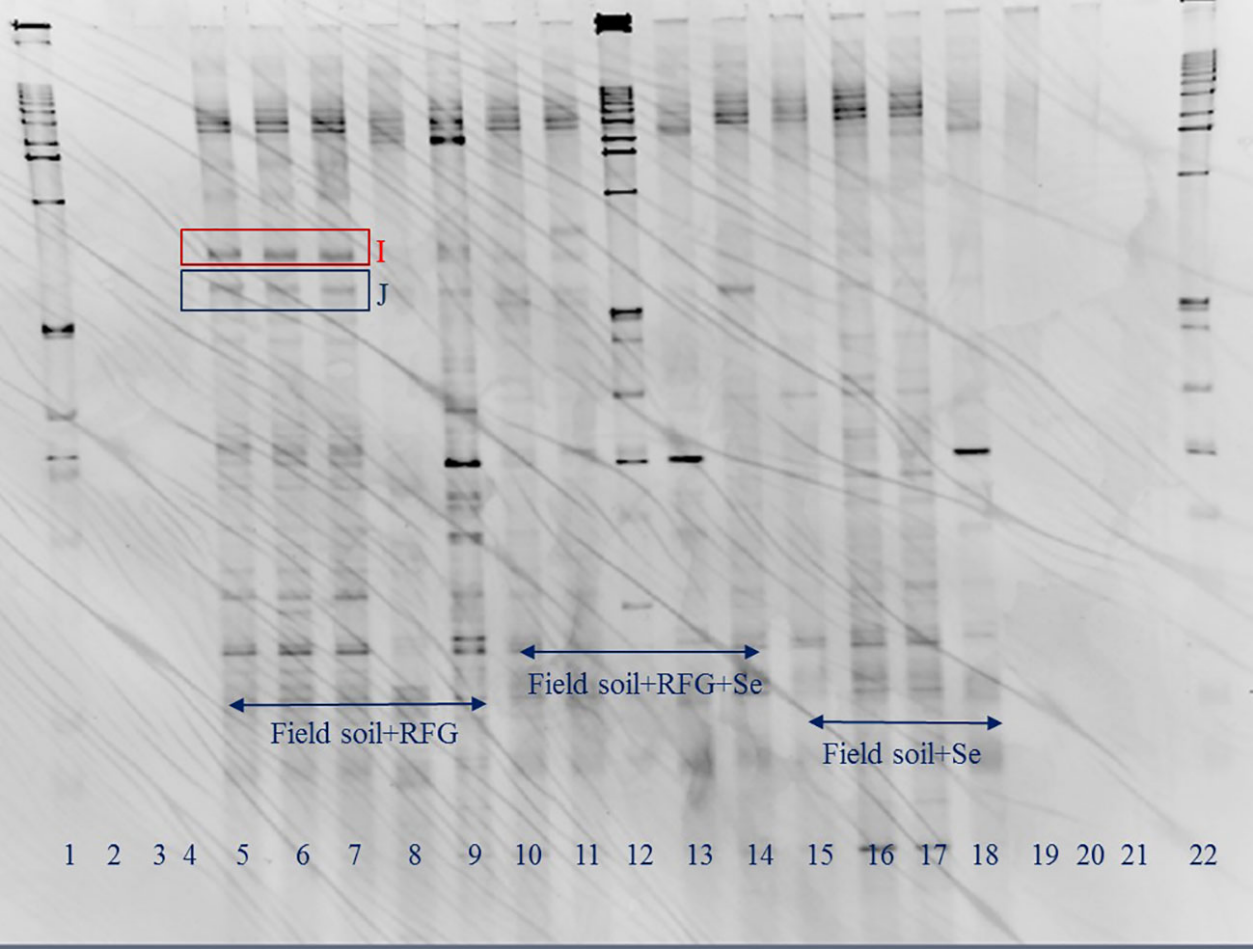
Effects of Se on microbial community in topsoil of a soil-rabbitfoot grass system. DGGE analysis of 16S rDNA fragments amplified from rhizosphere DNA taken from the rabbitfoot grass soil without Se treatment (lanes 5 to 9), the rabbitfoot grass soil with Se treatment (lanes 10 to 14), and the unvegetated soil with Se treatment (lanes 15 to 18). Lanes 1, 12 and 22: 1 kb ladders. Bands excised, cloned, sequenced and identified as: I: Uncultured Alphaproteobacterium Clone, J: Uncultured Bacterium Clone.

For samples to be cloned, PCR products were purified using Wizard SV Gel PCR Clean-Up System (Promega, Madison, WI) to remove excess nucleotides and primers. Purified PCR products were cloned into pGEM-T Easy vector (Promega), transformed into CaCl2-treated competent cells (*Escherichia coli* DH5 α), and screened by blue/white screening on Luria-Bertani (LB) agar plates containing ampicillin, X-gal, and IPTG. Plasmids were isolated from *E. coli* using standard protocols and a Wizard Plus SV Miniprep DNA Purification System kit (Promega). For DGGE plug sequences, purity and correct migrating positions of plasmids were confirmed by performing PCR on the plasmid using DGGE primers, and running the plasmid insert on DGGE gels opposite to PCR product of the specific excised DGGE bands. The DGGE band image (**Figure 2**) is a reversed greyscale image but without other enhancements. Clones having products with correct migration on a DGGE gel, as well as clones from the pure cultures, were sequenced at the Roy J. Carver Biotechnology Center, University of Illinois at Urbana-Champaign. Sequences were compared with the Genbank database using BLAST (Altschul et al., 1990).

### 2.2 Se volatilization by different bacterial strains isolated from the soil with rabbitfoot grass

For bacterial Se volatilization measurements, different bacterial strains isolated from the soil-plant system treated with 5 mg/kg of Se (SeO_4_) were first cultured in LB media containing 5 µg/ml of Se (SeO_4_) in order to acclimate bacteria to the Se treatments. LB broth was used as a medium for Se volatilization measurement based on a preliminary test that showed LB to be better than NB for Se volatilization measurement. Rates of Se volatilization by different soil bacterial strains were determined after inoculating each flask with an equal number of cells of each bacterial strain. Quantification of cell number was performed using Bull’s Eye counting calibrated tubes (MIDSCI, St. Louis, MO) per the manufacturer’s protocol (www.tpp-us.com/docs/protocol_tpp.pdf). A pellet containing 2 x 10^10^ CFU was re-suspended in 200 ml of LB broth containing 15 µg/ml Se (equal mixture of selenate, selenite and SeMet) in a 500 ml Erlenmeyer flask for Se-volatilization measurement.

#### 2.2.1 Microbial Se volatilization measurement

Culture flasks (500 ml), each with a double-hole stopper with inlet and outlet glass tubes, were shaken at 125 rpm. The air inlet tubing was attached to a 0.2 µm filter whereas the outlet tubing was connected to two gas washing bottles in series (Fisher Scientific, Chicago, IL). The first 500 ml gas washing bottle contained 200 ml trap solution (0.5M NaOH and 30% H_2_O_2_, 4:1, v/v), and the second 250 ml bottle was filled with 100 ml trap solution. The second gas washing bottle was connected to a vacuum pump via an airflow meter. The volatilization measurement was conducted in three replicates at room temperature (25 ± 1 °C), and the airflow rate was 70 L/h. The sampling period was 24 h. At the end of volatile Se sampling, flasks were removed from the shaker and placed on ice. The bacterial number in culture solution from each flask was determined immediately to determine bacterial growth in each flask during the 24-hour volatilization measurement. The volume of the trap solution in each gas washing bottle was measured, and a subsample of the trap solution was collected from each gas washing bottle and was stored at 4 °C until chemical analysis.

#### 2.2.2 Chemical analysis of total Se

To determine Se concentration, 4 ml of trap solution was transferred to a glass test tube and heated at 95 °C for 45 min to oxidize Se to selenate and remove H_2_O_2_ residue from the trap solution. Then, 4 ml of concentrated hydrochloric acid (TraceMetal grade) was added and heated again at 95 °C for 30 min to reduce selenate to selenite as determined by an atomic absorption spectrophotometer (Varian SpectrAA-220FS with VGA-77) (Lin et al., 2000).

### 2.3 Enhancement of Se volatilization in a soil-Indian mustard system inoculated with *Bacillus cereus*

Twelve pots were used for the study. The treatment and control groups included four pots (or four replicates) of soil-Indian mustard inoculated with the best Se-volatilizing bacterial strain (*B. cereus*), four pots of uninoculated soil with Indian mustard, and four pots containing only soil (unvegetated). Each pot contained a 500 g (DW) mixture of clay loam soil and fine sand. The experimental soil was fertilized by adding four “Miracle Gro” 6-12-6 fertilizer spikes. Indian mustard seeds (accession no. 173874) were obtained from the North Central Regional Plant Introduction Station (Ames, IA). After germination, Indian mustard plants grew for six weeks in a growth chamber where the temperature was 25°C, the relative humidity was approximately 70%, and light was continuous. The plants were irrigated daily.

Prior to the maturity stage (or flowering stage) the soil-Indian mustard pots in the growth chamber were inoculated with *B. cereus* that was isolated and identified from soil vegetated with rabbitfoot grass. LB broth was inoculated with the bacterial strain and incubated at 37 °C for 24 hours. The broth was centrifuged at 2400 x g for 15 minutes at 4 °C, and the cell pellet was resuspended in 1 x PBS. The number of bacterial cells added to each pot was determined using the same cell counting method (the Bull’s Eye method) that was described previously for the rabbitfoot grass inoculation experiment. Based on this count, approximately 2x10^10^ CFU (in 2 ml PBS solution) were added to each pot. The four vegetated pots that were not inoculated with bacteria and the four unvegetated soil pots received 2 ml of sterile PBS solution.

One week after the pots were inoculated with *B. cereus*, Se treatment was performed by adding sodium selenate (Na_2_SeO_4_) solution to the soil surface of all 12 pots to achieve a final soil Se concentration of 3 mg/kg (DW). After the Se treatment, each pot was irrigated with additional water for better Se distribution in the potting soil, and then each pot was enclosed in a Plexiglass volatilization chamber. Volatile Se was collected following the same method reported by Lin et al. (2000). The trap solution was replaced every 24 hours for a 7-day time period. Plants were irrigated with tap water on Days 3 and 5.

### 2.4 Statistical analysis

The volatilization experiments were carried out in a completely randomized design. Statistical analysis by one-way analysis of variance (ANOVA) was completed using the Statistical Analysis Software (SAS Inc. Cary, NC) for a completely randomized design. The level of significance (α) was 0.05.

## 3 Results

### 3.1 Effects of Se treatments on the soil microbial community of rabbitfoot grass

Rhizosphere soil samples cultured on nutrient agar plates yielded a diverse range of colony types compared with topsoil samples from the same pots. Colonies were grouped according to similarities in shape, size, color, and/or texture. From rhizosphere soils, 23 different colony types from the pots without Se treatment (or rabbitfoot grass pots without Se treatment) were visually identified, while 14 colony types were observed from the pots treated with Se (or rabbitfoot grass pots with Se treatment). However, the Se treatment had no effect on the number of colonies visually identified from topsoils. According to the 16S rDNA sequencing analysis, cultured pure bacterial isolates unique to particular conditions or soil regions were identified as *Pseudomonas* sp., *Paenibacillus* sp., *Bacillus* sp., *Pseudoxanthomonas* sp., *Agrobacterium* sp., *Methylobacterium* sp., *Streptomyces* sp., along with some unidentified uncultured bacterium clones (**Table 1**).

**Table 1:** Identification of bacterial isolates from rabbitfoot grass soil (topsoil and rhizosphere) with Se treatment or without Se treatment.

Strain	Genbank Accession	Top Soil		Rhizosphere Soil	
		With Se treatment	Without Se treatment	With Se treatment	Without Se treatment
*Bacillus cereus*	EU855219	–	–	99%	99%
*Pseudomonas putida*	AY958233	–	–	99%	–
*Bacillus megaterium*	EU880506	99%^†^	99%	99%	99%
*Pseudomonas teessidea*	AM419154	–	–	100%	–
*Paenibacillus barcinonensis*	AJ716019	–	–	99%	–
*Bacillus subtilis*	EU257452	–	–	99%	–
*Methylobacterium Species*	DQ838528	–	–	99%	–
*Streptomyces graminearus*	EF371437	–	–	99%	–
Uncultured Bacterium Clone	DQ337029	98%	–	98%	–
*Streptomyces albogriseolus*	AJ494865	99%	–	–	–
*Pseudoxanthomonas mexicana*	AF273082	–	–	–	99%
*Paenibacillus lautus*	AJ491842	–	–	–	99%
Uncultured Bacterium Clone	DQ351909	–	–	–	94%
*Bacillus acidiceler*	DQ374637	–	–	–	98%
*Bacillus samanii*	EF036537	–	–	–	100%
*Agrobacterium tumifaciens*	EF217305	–	100%	–	–
*Bacillus Species*	AM177061	–	99%	–	–
*Pseudomonas Species*	U781733	–	–	–	–

^†^ % indicates identity of rDNA sequence over 700 bp query.

In order to avoid potential culture-based limitations that might preclude the identification of any nonculturable bacteria present in the soil-rabbitfoot grass system, PCR-DGGE was also conducted on genomic DNA isolated from the rabbitfoot grass soil with Se treatment and the rabbitfoot grass soil without Se treatment, and the unvegetated soil with Se treatment. The PCR-DGGE results showed significant alteration in the rhizosphere bacterial community as several differences in the DGGE banding pattern were observed (**Figure 1**). The numbers of bands detected from these three treatments were 17, 8, and 9, respectively. This difference in the banding pattern was statistically significant (p<0.01) between the rabbitfoot grass soil with Se treatment versus the rabbitfoot grass soil without Se treatment. Sorenson’s coefficient calculation for DGGE gel determined 64-76% similarity coefficient (Cs) among the treatments (**Supplementary Figure S1**). A higher similarity index in rhizosphere soil demonstrated that more Se-tolerant species were associated with rabbitfoot grass roots. The unique dominant bands in lane 8-13 (**Figure 1**) were excised, cloned, and sequenced under the assumption that these microbial species better represented the rhizosphere of rabbitfoot grass treated with Se. The 16SrDNA sequencing of those selected dominant DGGE bands from “RFG without Se” rhizosphere included *Niastella* sp., and nonculturable bacterium clone, whereas “RFG with Se” included uncultured Xanthomonadales and Alphaproteobacterium clone. Two unique DGGE bands were observed in the topsoil of rabbitfoot grass without the Se treatment, and were identified noncultured Alphaproteobacterium clone, and uncultured bacterium clone (**Table 2**; **Figure 2**).

**Table 2:** Identification of selected unique DGGE bands detected in genomic DNA extracted from rabbitfoot grass soil fractions with Se treatment or without Se treatment.

Strain	Genbank Accession	Top Soil		Rhizosphere Soil	
		With Se treatment	Without Se treatment	With Se treatment	Without Se treatment
Uncultured Xanthomonadales Bacterium Clone	EU404026			100%	
Uncultured Bacterium Isolate	EF599659			100%	
*Niastella* sp.	EU877263				99%
Uncultured Alpha Proteobacterium Clone	EF188320	99%			97%
Uncultured bacterium Clone	EU869747	98%			

% indicates identity of rDNA sequence over 700 bp query.

### 3.2 Rates of Se volatilization by the isolated bacterial strains

**Figure 3:**
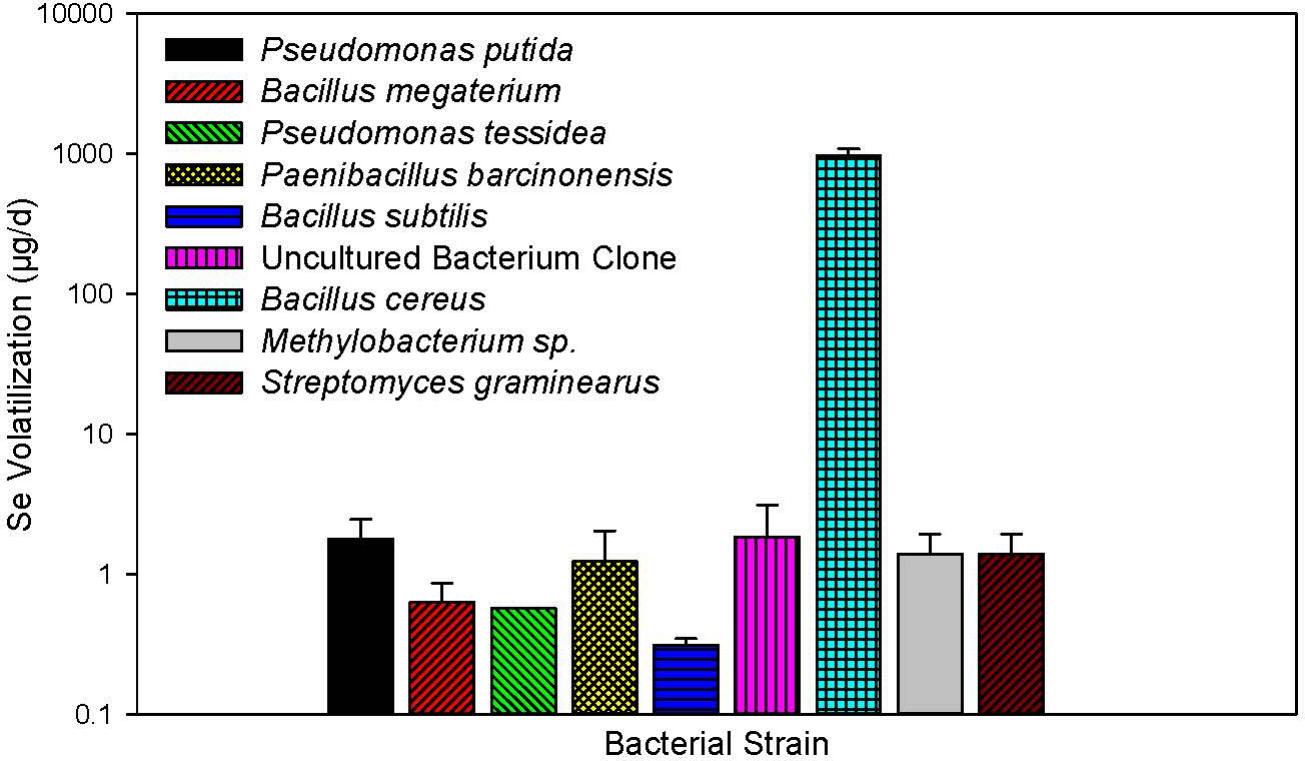
Selenium volatilization rates among bacterial strains isolated from the soil-rabbitfoot grass system. Microbial volatilization measurements conducted over 24 hours demonstrated *B. cereus* as the best Se volatilizing species at p < 0.05. The data in the figure are means and standard deviation (n=3).

Selenium volatilization measurements of nine different bacterial isolates from rabbitfoot grass soils (both topsoil and rhizosphere) with Se treatment revealed that *B. cereus* had the highest Se volatilization rate among tested strains (**Figure 3**; p<0.001). *B. cereus* volatilized 965 ± 116 µg Se per flask per day, followed by approximately 0.5-2 µg Se per flask per day by each of the other bacterial strains (**Figure 3**). The Se mass balance recovery rate in each volatilization flask was 96% (**Table 3**).

**Table 3:** Selenium mass balance in the *Bacillus cereus* volatilization experiment.

Se Mass at time (h) = 0		Se Mass at time (h) = 24		Se Recovery
Source	Total Se	Source	Total Se	
Pre-cultured cells (5 μg Se/ml)	45.10 μg	Se Volatilization	964.82 μg	
Se treatment (15 μg Se/ml)	3000 μg	Se in culture solution	1945.82 μg	
Total inputs	3045.10 μg	Total outputs	2913.18 μg	95.6%

The total volume of bacterial culture solution was 200 ml.

### 3.3 Effects of *B. cereus* inoculation on Se volatilization in the soil-Indian mustard system

**Figure 4:**
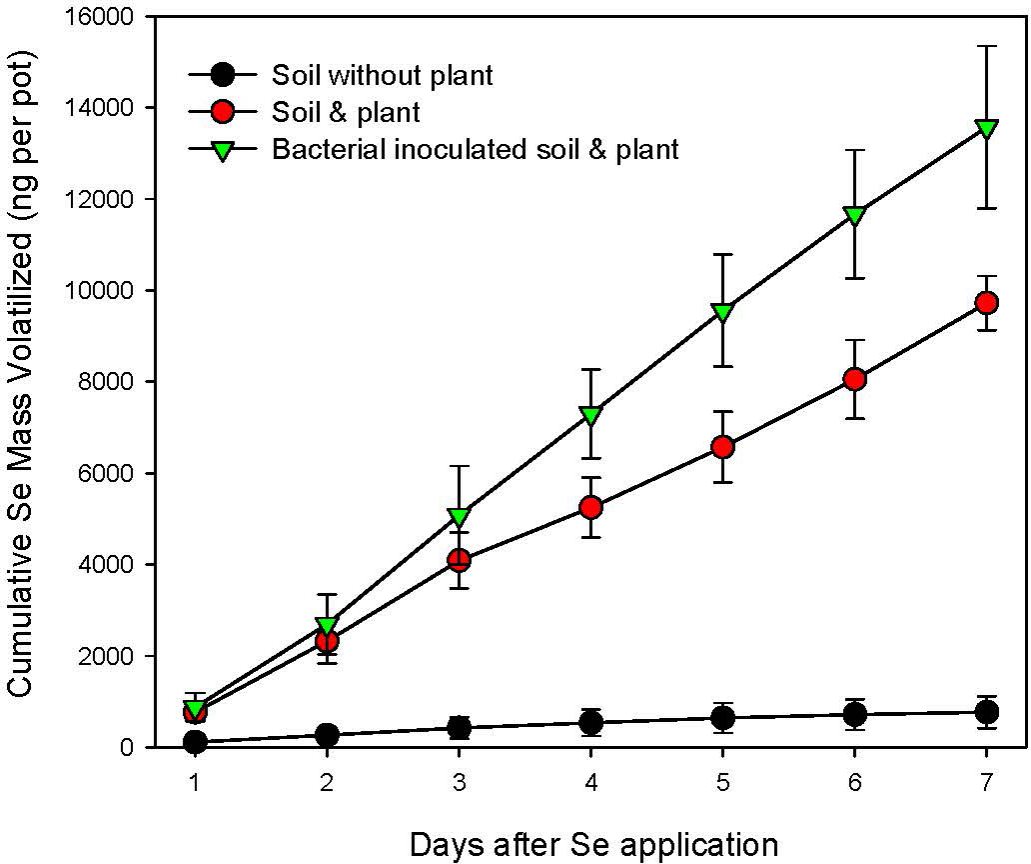
Cumulative selenium mass volatilized from the soil-Indian mustard (*B. juncea*). The data are means and standard deviations (n=4).

Inoculation of Indian mustard vegetated soil with the best Se volatilizing bacterial strain identified in previous experiments, *B. cereus*, resulted in a significant (p<0.05) increase in Se volatilization during a continuous 7-day experimental period, compared to the soil-plant system without the added *B. cereus*. The cumulative Se mass volatilized during this experiment is shown in **Figure 4**. The cumulative Se mass volatilized from each pot increased with time from Day 1 to Day 7. Indian mustard plant significantly (p<0.05) enhanced the amount of Se mass volatilized from the soil-plant system, compared with un-vegetated soil. Further, a significant increase in Se volatilization in the soil-Indian mustard systems was also observed after inoculation with *B. cereus*. The total amount of Se mass volatilized from the unvegetated soils was unchanged significantly during the 7-day experiment. The addition of the rabbitfoot grass-associated *B. cereus* strain to the soil-Indian mustard system led to a significant enhancement of Se volatilization from Se-treated soils (**Figure 4**).

## 4 Discussion

Molecular microbiological techniques based on the approximately 1500 bp 16S ribosomal DNA (rDNA) molecule were used in this study. The presence of rDNA regions that are conserved among all bacterial species along with regions that vary among different species allows the rDNA gene to be used for assessment of microbial communities without culturing, as well as for identification of unknown isolates (Olsen et al., 1986). The strains identified in this study were tested for their Se volatilization ability, and the strain *B. cereus* with superior ability to volatilize Se in the rabbitfoot grass model was inoculated into a soil-Indian mustard system to determine any enhancement in Se-volatilization in that model system. This study revealed that plant-microbe interactions in soils is very important, and rhizosphere soil bacteria play an important or major role in Se volatilization in plant-soil systems. Further, the rate of Se volatilization is also dependent on the chemical form of Se, concentration of Se, and the plant species involved. Similar results have been reported by several previous studies (Terry and Zayed, 1994; de Souza et al., 1998; Pilon-Smits et al., 1999). For example, in a treatment wetland vegetated with rabbitfoot grass, biogenic volatilization of Se removed 35-49% of the monthly Se input during late spring and early summer under the field conditions (Lin and Terry, 2003).

In order to define the plant-microbe interaction in Se volatilization in soil-rabbitfoot grass system, microbial community analysis from soil-rabbitfoot grass system was performed using both culture-dependent and molecular approaches. Fewer microbial species were detected using culture-based microbiological techniques in Se-treated soils (5 mg Se/kg), indicating that Se-contamination may significantly impact the microbial community of soil-rabbitfoot grass system. This was also illustrated with the reduction in the DGGE band number, because some Se-susceptible species diminished in the Se-contaminated soils. A higher similarity index of about 64-76% (**Supplementary Figure S1**) in rhizosphere soil demonstrated that more Se-tolerant species were associated with rabbitfoot grass roots.

Several bacterial strains isolated from soil-rabbitfoot grass root systems have been reported. For example, *Pseudomonas putida* was isolated from a Se-contaminated mine site and *Bacillus subtilis* from seleniferous agriculture drainage (Dungan and Frankenberger, 1999; Knotek-Smith et al., 2006; Dhanjal and Cameotra, 2010). In the present study *P. putida* and *B. subtilis* were also isolated from rabbitgrass rhizosphere soil treated with Se (**Table 1**). However, this study also observed that *B. cereus* is the rabbitfoot grass-associated soil bacterial strain with the highest Se volatilization (**Figure 3**). *B. cereus* is a Gram-positive rod-shaped bacterium and is often associated with contaminated grain. It has also been previously isolated from heavy metal-contaminated soil (Ellis et al., 2003). All bacterial strains used for Se volatilization in this study were isolated from the rhizosphere, although some of them were also isolated from the topsoil. Incubation of these samples at 37 °C instead of ambient temperature may have led to a different constellation of isolates than would have occurred otherwise; nevertheless, *B. cereus* outperformed all these isolates at Se volatilization. Thus, this study is one of few that evaluated the potential of *B. cereus* for Se volatilization in a soil-plant system.

In this study the bacterial number was determined using a calibrated counting chamber, rather than optical density (OD) measurement, largely due to the color change of bacterial media from the formation of reddish elemental Se. The initial bacterial number for the Se volatilization experiment was 2 x 10^10^ CFU/200 ml or 10^8^ CFU/ml (**Supplementary Table S1**). The volatilization experiment showed *B. cereus* to have the highest Se volatilization ability, as it volatilized approximately 964 µg Se from 200 ml culture solution treated with 15 µg/ml of Se over 24 h. Variation in the growth of bacterial cells was observed by the end of 24 h. Some of the strains did not grow as well as *B. cereus* but the others had similar or even higher growth rates than *B. cereus*; nevertheless, *B. cereus* volatilized the largest amount of Se among all the strains. The cell suspension after 24 h of Se volatilization may have had some endospores in total cell volume that were no longer metabolically active (**Supplementary Table S1**). Endospores would have been counted, but they would not have been relevant for Se volatilization measurement if not bioactive.

A significant increase in Se volatilization was observed in the soil-Indian mustard system inoculated with *B. cereus* compared to the control. The total amount of Se mass volatilized from the unvegetated soils did not show significant changes during the 7-day experimental period. However, the presence of Indian mustard plants significantly increased Se volatilization, compared to unvegetated soils. The soil-Indian mustard system inoculated with *B. cereus* showed a significant difference in Se volatilization as soon as 4 days after the soil Se treatment (**Figure 4**). Thus, addition of rabbitfoot grass-associated *B. cereus* strain to the soil-Indian mustard system could be important for the enhancement of Se volatilization from Se-contaminated environments. An earlier laboratory study by de Souza et al. (1999) also showed that rhizosphere bacteria could overcome the rate limitation of Se volatilization from selenate (de Souza et al., 1998; Terry et al., 2000). We speculate that *B. cereus* might have played an important role in overcoming the biotransformation limit from selenate to selenite, and thus enhanced the reduction of selenate (the chemical form of Se supplied in the soil) to selenite that can be readily volatilized by soil microbes. This is particularly important for Indian mustard because it is a good Se accumulator species, but not an excellent plant species for Se volatilization. Zhang et al. (2024) reported that *B. cereus* can significantly increase the biomass production and Se accumulatio of *B. napus*. Thus, future studies need to be conducted to determine the ability of *B. cereus* in biotransformation and speciation of Se in rhizosphere soil of Indian mustard.

The presence of vegetation significantly increased Se volatilization compared with bare soil (or un-vegetated soil). The inoculation with *B. cereus* significantly increased levels of Se volatilization from soil-Indian mustard system. Our results indicated that there was an overall increase in Se volatilization between the inoculation treatment and the control (i.e., the soil-Indian mustard system without the bacterial inoculation treatment). The increase may be due in part to the change of soil *B. cereus* biomass.

## Data Availability

The datasets presented in this study can be found in online repositories. The names of the repository/repositories and accession number(s) can be found in the article/**Supplementary Material**.
