# Supplementary material for: Enhancement on selenium volatilization for phytoremediation: role of plant and soil microbe interaction
Source: Front Plant Sci. 2024 Dec 23;15:1504528. doi: 10.3389/fpls.2024.1504528 (PMC11700991; doi:10.3389/fpls.2024.1504528)
Supplement: Supplementary file 1 [file DataSheet1.docx]

# Supplementary Material

Enhancement on Selenium Volatilization for Phytoremediation: Role of Plant and Soil Microbe Interaction

Ranju R. Karna^1^, Samantha T. Kumara^1^, Vance J. McCracken^2*^, Thomas J. Fowler^2^, Zhi-Qing Lin^1,2*^

^1^Department of Environmental Sciences, Southern Illinois University Edwardsville, Edwardsville, Illinois 62026

^2^Department of Biological Sciences, Southern Illinois University Edwardsville, Edwardsville, Illinois 62026

^*^Corresponding author emails: [vmccrac@siue.edu](mailto:vmccrac@siue.edu) (VJM); [zhlin@siue.edu](mailto:zhlin@siue.edu) (ZQL)

# Data availability statement

The original contributions are presented in the article and supplementary material, and the additional data are made available by the authors at: <https://www.siue.edu/~zhlin/Press/Karna%20et%20al.%202024%20Front%20Plant%20Sci%20Data%20File.pdf>.

**SI Figure 1.** Sorensen’s coefficient (CS) of rhizosphere soil from each treatment. Percentage of similarities for the unvegetated soil without Se treatment (or Soil), the unvegetated soil treated with 5 mg kg^-1^ Se (or Soil + Se), and the rabbitfoot grass soil with 5 mg kg^-1^ Se treatment (or Soil+Se+RFG) groups after 4 weeks. Sorenson’s similarity index was used to compare average percent similarities of DGGE banding patterns (based on the average number of bands in common) for different samples within each treatment group.

**SI Table 1.** Bacterial growth in 200 ml cultural solution treated with 15 µg Se mL^-1^ in a 500 ml flask during a 24-h Se volatilization measurement at the room temperature.

| **Strain** | **Initial CFU/200 ml** | **Final CFU/200 ml** |
| --- | --- | --- |
| *Pseudomonas putida* | 2 x 10^10^ | 6.93 x 10^11^ |
| *Bacillus megaterium* | 2 x 10^10^ | 3.54 x 10^11^ |
| *Pseudomonas teessidea* | 2 x 10^10^ | 2.6 x 10^11^ |
| *Bacillus subtilis* | 2 x 10^10^ | 7.2 x 10^10^ |
| Uncultured Bacterium Clone | 2 x 10^10^ | 3.0 x 10^10^ |
| *Bacillus cereus* | 2 x 10^10^ | 5.48 x 10^11^ |
| *Methylobacterium sp.* | 2 x 10^10^ | 2.62 x 10^10^ |
| *Streptomyces graminearus* | 2 x 10^10^ | 2.12 x 10^10^ |


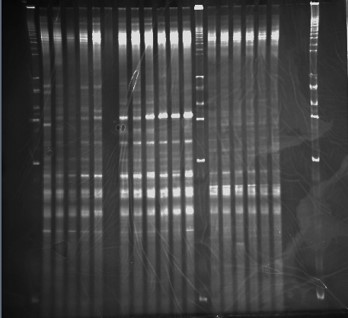


**SI Figure 2**. The full scan of the entire original gel image of Figure 1 - Effects of Se on the microbial community in the rhizosphere of a soil-rabbitfoot grass system.


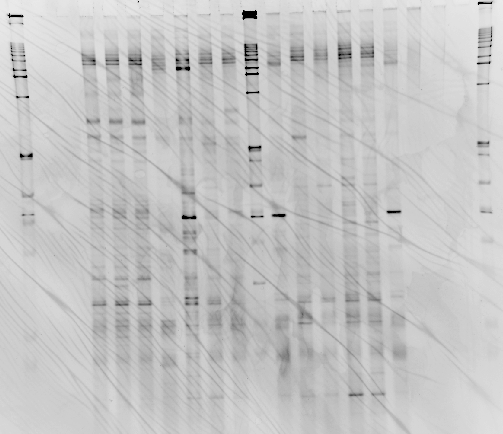


**SI Figure 3**. The full scan of the entire original gel image of Figure 2 - Effects of Se on microbial community in topsoil of a soil-rabbitfoot grass system.
